# Inflammatory and Genetic Determinants of Functional Status in HFpEF: The Role of Circulating IL-6 and *IL6* Polymorphisms

**DOI:** 10.3390/biomedicines14081783

**Published:** 2026-08-07

**Authors:** Neda Ćićarić, Sanja Matić, Natasa Djordjevic, Tijana Marković, Marijana Stanojević-Pirković, Đorđe Stevanović, Nina Uraković, Željko Ivošević, Lidija Stojanović, Goran Davidović, Marija Popović, Jelena Vučković-Filipović, Ivan Simić, Dejan Baskić, Vladimir Zdravković

**Affiliations:** 1 Clinic for Cardiology, University Clinical Center Kragujevac, 34000 Kragujevac, Serbia; nedaa.cicaric@gmail.com (N.Ć.); djordje.stevanovic.kg@gmail.com (Đ.S.); n.urakovic@gmail.com (N.U.); likistojanovic@gmail.com (L.S.); medicusbg@yahoo.com (G.D.); marijapopovickg@gmail.com (M.P.); jelenavufi@gmail.com (J.V.-F.); ivansimickg@gmail.com (I.S.); vladazdrav@gmail.com (V.Z.); 2 Department of Pharmacology and Toxicology, Faculty of Medical Sciences, University of Kragujevac, 34000 Kragujevac, Serbia; natashadj2002@yahoo.com; 3 Department of Pharmacy, Faculty of Medical Sciences, University of Kragujevac, 34000 Kragujevac, Serbia; tianafeels@gmail.com (T.M.); dejan.baskic@gmail.com (D.B.); 4 Center for Laboratory Diagnostics, University Clinical Center Kragujevac, 34000 Kragujevac, Serbia; marijanas14@gmail.com; 5 Department of Medical Biochemistry, Faculty of Medical Sciences, University of Kragujevac, 34000 Kragujevac, Serbia; 6 Department of Internal Medicine, Faculty of Medical Sciences, University of Kragujevac, 34000 Kragujevac, Serbia; zeljkoivosevic274@gmail.com; 7 Clinic for Endocrinology, Diabetes and Metabolic Diseases, University Clinical Center Kragujevac, 34000 Kragujevac, Serbia; 8 Public Health Institute, 34000 Kragujevac, Serbia

**Keywords:** HFpEF, interleukin-6, inflammation, genetic polymorphism, functional capacity, NYHA class, 6MWT, Borg scale

## Abstract

**Background/Objectives**: Heart failure with preserved ejection fraction (HFpEF) is a heterogeneous syndrome characterized by systemic inflammation and impaired functional capacity. Interleukin-6 (IL-6) is implicated in HFpEF pathophysiology, but the influence of IL6 genetic variability on its clinical impact remains unclear. This study investigated the associations of circulating IL-6, IL6 polymorphisms, and functional severity in HFpEF. **Methods**: This single-center observational study included 110 clinically stable HFpEF patients. Clinical, laboratory, and functional data, including C-reactive protein (CRP), IL-6 serum levels were collected. The neutrophil-to-lymphocyte ratio and platelet-to-lymphocyte ratio were calculated from complete blood count, while *IL6* polymorphisms rs1800795 and rs1818879 were genotyped using TaqMan assays. Functional status was assessed using NYHA classification, the six-minute walk test (6MWT), and the Borg dyspnea scale. **Results**: Among carriers of the rs1800795 G/C+C/C genotype, elevated IL-6 was associated with higher odds of advanced symptoms (NYHA III; OR = 6.08, 95% CI 1.45–25.45, *p* = 0.014) and reduced exercise capacity (6MWT; OR = 6.55, 95% CI 1.60–26.72, *p* = 0.009). The combined effect of elevated IL-6 levels and the rs1800795 genotype was also associated with greater dyspnea burden, as assessed by the Borg scale (OR = 1.17, 95% CI 1.02–1.34, *p* = 0.029). In multivariable analysis, the NYHA and 6MWT models additionally retained NLR as an independent predictor, while the Borg model included advanced age, pulmonary hypertension, and the combined effect of elevated IL-6 levels and the rs1800795 genotype. **Conclusions**: The observed combined effect of elevated IL-6 levels and the rs1800795 genotype on symptom burden and functional impairment suggests that combined inflammatory and genetic profiling may improve severity assessment in HFpEF.

## 1. Introduction

Heart failure (HF) with preserved ejection fraction (HFpEF) represents a growing and clinically challenging syndrome, accounting for nearly half of all HF cases and characterized by substantial heterogeneity and limited therapeutic options [1,2,3]. This trend is mainly driven by population aging, as well as by rising obesity rates and associated cardiometabolic comorbidities [4,5]. Despite its clinical relevance, HFpEF remains underrecognized in early stages [6]. Consequently, many patients initially present with decompensation requiring hospitalisation, which is associated with in-hospital mortality of approximately 6.5% and high five-year mortality [6,7].

Contemporary pathophysiological concepts describe HFpEF as a systemic, multiorgan syndrome in which chronic low-grade inflammation, driven by comorbid conditions, induces endothelial dysfunction and coronary microvascular impairment [2,8,9]. This inflammatory milieu promotes adverse myocardial remodelling, including hypertrophy, increased stiffness, and impaired diastolic function, ultimately giving rise to the characteristic hemodynamic and clinical manifestations of HFpEF [2,8]. In this context, inflammation is increasingly regarded not merely as an associated phenomenon, but as a central mechanistic driver that links comorbid burden to structural and functional myocardial alterations in HFpEF.

Interleukin-6 (IL-6) is an important mediator within this inflammatory network. As a pleiotropic cytokine, IL-6 is involved in endothelial activation, regulation of extracellular matrix turnover, oxidative stress, and cardiomyocyte hypertrophy and fibrosis, thereby directly connecting systemic inflammation with adverse myocardial remodelling [10,11]. Elevated circulating IL-6 levels have been associated with increased symptom burden, impaired functional capacity, and worse outcomes in HF, indicating its relevance to HFpEF pathophysiology [8]. Collectively, these findings support the concept that IL-6-mediated inflammatory signalling may represent an important mechanistic link between systemic comorbidities and myocardial dysfunction in HFpEF.

Interindividual variability in inflammatory activation, including IL-6 signalling, is partly determined by genetic background. Polymorphisms within the *IL6* gene can influence gene expression and circulating cytokine levels, thereby modulating the intensity and chronicity of inflammatory responses [12]. Such genetic variation has been linked to differences in cardiometabolic risk, vascular dysfunction, and clinical outcomes across a range of chronic inflammatory and cardiovascular conditions [13]. Given that HFpEF is strongly driven by inflammation-mediated microvascular and myocardial dysfunction, *IL6* polymorphisms represent biologically plausible contributors to disease heterogeneity.

However, the mechanisms underlying interindividual variability in inflammatory burden and clinical expression in HFpEF remain insufficiently defined [14]. A better understanding of these genetic influences may help explain the substantial interindividual variability in inflammatory burden and clinical expression observed among patients with HFpEF. Such knowledge has the potential to refine current pathophysiological models, improve risk stratification, and support the development of more personalized therapeutic approaches.

Accordingly, this study aimed to investigate the individual and combined predictive contributions of systemic inflammatory activation and IL6 genetic polymorphisms to functional impairment in patients with HFpEF, encompassing symptom severity, exercise capacity, and perceived exertional dyspnea.

## 2. Materials and Methods

### 2.1. Study Design

This study was designed as a single-center, non-interventional, observational cross-sectional study involving patients diagnosed with HFpEF who were hospitalized at the Cardiology Clinic of the University Clinical Center Kragujevac, Serbia, between November 2023 and November 2024.

The diagnosis of HFpEF was established according to current European Society of Cardiology guidelines [15], requiring: (1) symptoms and signs of HF; (2) left ventricular ejection fraction ≥50%; and (3) objective evidence of structural and/or functional cardiac abnormalities consistent with diastolic dysfunction or elevated left ventricular filling pressures, including increased natriuretic peptide levels.

The study protocol was approved by the institutional Ethics Committee (Decision No. 01/23-481, 23 October 2023), and all patients provided written informed consent.

### 2.2. Study Population

The study included 110 adult patients with HFpEF, enrolled between November 2023 and November 2024 at the University Clinical Center Kragujevac (Serbia). All participants were included during a clinically stable phase of the disease.

Participants were eligible for inclusion if they were aged 18 years or older, had a confirmed diagnosis of HFpEF according to established criteria [15], and had clinically stable disease. Clinical stability was defined as a period of at least four weeks without hospitalization due to heart failure decompensation, urgent specialist consultation requiring intensification of heart failure therapy, unplanned outpatient visits prompted by worsening symptoms, or patient-initiated escalation of diuretic treatment. Patients were excluded if they declined to provide informed consent, had experienced changes in cardiovascular therapy within the four weeks preceding assessment, or had an acute or recent infection during the previous four weeks. Additional exclusion criteria included acute coronary syndrome or cerebrovascular events within the preceding three months, systemic connective tissue diseases, neurological or psychiatric disorders, inflammatory bowel disease, and specific cardiac conditions such as infiltrative cardiomyopathies (including sarcoidosis and amyloidosis), primary hypertrophic cardiomyopathy, arrhythmogenic right ventricular cardiomyopathy, Takotsubo cardiomyopathy, or active myocarditis. Patients were also excluded if they had reduced pulmonary function (forced vital capacity < 60% of the predicted value within the past year), severe anemia (hemoglobin < 80 g/L), terminal-stage malignancy, end-stage renal impairment (estimated creatinine clearance < 15 mL/min/1.73 m^2^), or significant valvular heart disease [16].

### 2.3. Data Collection and Clinical Assessment

Clinical, demographic, and medical history data, including age, sex, and comorbidities, were obtained through structured interviews and verified using electronic medical records. The burden of comorbidities was quantified using the Charlson Comorbidity Index (CCI).

Laboratory analyses included standard biochemical parameters and markers of systemic inflammation (C-reactive protein (CRP) and IL-6), measured under standardized conditions at the Department of Laboratory Diagnostics. CRP and IL-6 concentrations were dichotomized using the upper reference limits of the clinical laboratory performing the analyses (>5 mg/L and >7 pg/mL, respectively). Neutrophil-to-lymphocyte ratio (NLR) was calculated as the ratio of absolute neutrophil/granulocyte count to absolute lymphocyte count, with the cut-off at 3 [17]. Platelet-to-lymphocyte ratio (PLR) was calculated as the ratio of platelet count to absolute lymphocyte count, with the cut-off at 150 [17].

Peripheral volume overload was quantitatively assessed using a standardized pitting edema scale [18]. The scale classifies edema severity based on the depth and duration of indentation following the application of pressure with the fingertips. The assessment was performed during the physical examination at the time of patient inclusion in the study.

Anthropometric and body composition measurements were performed at baseline. Body height and weight were measured using standardized methods, and body mass index (BMI) was calculated. Waist circumference (WC) was measured according to standardized anatomical landmarks and categorized using European cut-off values (≥94 cm for men and ≥80 cm for women) to define abdominal obesity [19]. Body composition, including total body fat percentage (BF%) and visceral fat (VF), was assessed using bioelectrical impedance analysis with a TANITA BC-547 scale (Tanita Corporation, Tokyo, Japan), in accordance with the manufacturer’s instructions [20].

Functional status was assessed using the New York Heart Association (NYHA) classification [21], based on patient-reported symptoms and evaluated by experienced cardiologists. Additionally, functional capacity was evaluated using the standardised six-minute walk test (6MWT), performed in accordance with international guidelines [22]. Predicted 6MWT distance was calculated using the reference equations proposed by Enright and Sherrill, taking into account sex, age, height, and body mass [23]. The lower limit of normal (LLN) was defined as the predicted value minus 153 m for men and minus 139 m for women. Accordingly, patients were categorized into two groups: satisfactory walking distance/normal functional capacity (achieved 6MWT distance ≥ LLN) and low walking distance/reduced functional capacity (achieved 6MWT distance < LLN).

Perceived exertional dyspnea during the test was quantified using the modified Borg scale, and for analytical purposes, values were categorized into lower (0–4) and higher (5–10) symptom burden groups [24].

### 2.4. DNA Extraction and Genotyping

Genomic DNA was isolated from 200 μL of whole blood collected in EDTA tubes using the PureLink GeneJET Genomic DNA Purification Kit (Thermo Scientific, Waltham, MA, USA), following the manufacturer’s protocol. DNA concentration and purity were evaluated by UV–Vis spectrophotometry on a BioTek Epoch Microplate Spectrophotometer (Agilent Technologies, Santa Clara, CA, USA), based on standard absorbance readings at 260 nm and 280 nm. DNA samples with A260/A280 ratios within the range of 1.7–1.9 were considered of adequate quality and were selected for subsequent genotyping.

Genotyping of *IL6* single-nucleotide polymorphisms (rs1818879 and rs1800795) was performed using TaqMan allelic discrimination assays on a Bio-Rad CFX96 Real-Time PCR Detection System (Bio-Rad, Hercules, CA, USA). Predesigned, commercially available TaqMan SNP Genotyping Assays (20×; Applied Biosystems, Foster City, CA, USA) were used for each polymorphism (C__26518729_10 for rs1818879 and C___1839697_20 for rs1800795). Target regions were amplified using TaqMan Genotyping Master Mix (2×; Applied Biosystems, Foster City, CA, USA).

PCR reactions were set up in a final volume of 20 μL containing 10 μL of TaqMan Genotyping Master Mix (2×), 0.5 μL of the appropriate TaqMan genotyping assay (20×), 0.5 μL of deionized/nuclease-free water, and 9 μL of genomic DNA (Applied Biosystems, Waltham, MA, USA). For quality assurance, deionized/nuclease-free water was used instead of DNA as a non-template control in each run to exclude contamination. Genotype calls were assessed independently by two investigators, and replicate analyses of selected samples showed complete concordance.

### 2.5. Statistical Analysis

Statistical analysis was performed using SPSS version 25.0 (IBM Corporation, Armonk, NY, USA) and STATA version 15 (STATA Corporation, College Station, TX, USA). Continuous variables were expressed as median (interquartile range) and compared using the Mann–Whitney U test, while categorical variables were presented as counts (percentages) and compared using the chi-square (χ^2^) test. Univariable and multivariable logistic regression analyses were performed to assess factors associated with advanced functional status (NYHA class III), reduced functional capacity as assessed by the 6MWT, and higher Borg scale categories. Variables included in the multivariable models were selected based on clinical relevance and the results of the univariable analyses. Results were expressed as odds ratios (OR) with 95% confidence intervals (CI).

For the predictive analyses, a combined IL-6–rs1800795 variable was constructed as the product of dichotomized IL-6 status (0: ≤7 pg/mL; 1: >7 pg/mL) and the dominant rs1800795 genetic model (0: G/G; 1: G/C+C/C). Accordingly, a value of 1 represented the joint presence of elevated IL-6 levels and the G/C+C/C genotype group, whereas a value of 0 represented all other combinations. This variable was entered into the multivariable models as a single predictor to assess the combined predictive contribution of IL-6 and the rs1800795 genotype rather than formal biological effect modification.

Multicollinearity among the inflammatory predictors included in the multivariable models was assessed using tolerance and variance inflation factor (VIF). Tolerance values >0.2 and VIF values < 5 were considered indicative of the absence of problematic multicollinearity. No evidence of problematic multicollinearity was detected, as all tolerance values were >0.47 and all VIF values were <2.1. To minimize the risk of overfitting, the number of variables retained in the final multivariable models was intentionally restricted. The final models included three variables, corresponding to approximately 9.7, 8.3, and 12.7 outcome events per variable in the NYHA, 6MWT, and Borg models, respectively. Firth’s penalised likelihood logistic regression was applied in cases of zero-cell frequencies. Model calibration was assessed using the Hosmer–Lemeshow test, while discriminative performance was evaluated using receiver operating characteristic (ROC) curve analysis and expressed as area under the curve (AUC). A *p*-value < 0.05 was considered statistically significant.

## 3. Results

### 3.1. Baseline Patients’ Characteristics

The study population consisted of 110 subjects, whose baseline demographic and clinical characteristics have been previously reported [25]. Briefly, there was a predominance of older patients, both sexes equally distributed, high burden of cardiometabolic comorbidities was present (most commonly arterial hypertension, hyperlipidemia, and diabetes mellitus), and approximately half of the patients were obese. Peripheral edema was present in most patients, commonly corresponding to mild severity (pitting edema grade 1). The study population consisted exclusively of patients with NYHA functional class II and III heart failure, with nearly two-thirds of participants classified as NYHA class II. Reduced functional capacity, defined as a decreased predicted 6MWT distance, was observed in 22.5% of patients. Additionally, approximately one-third of participants reported severe dyspnea, as indicated by a Borg score greater than 5.

Main demographic, anthropometric clinical features and laboratory parameters of HFpEF patients classified according to functional status in terms of NYHA classification, predicted 6MWT distance and Borg scale are presented in Table 1. Results of univariable logistic regression analyses are presented in Supplementary Table S1.

Advanced age was associated with worse functional status across all evaluated outcomes: patients aged ≥75 years were more frequently classified as NYHA III and reported higher Borg scores, while a similar trend was observed for reduced 6MWT performance. Logistic regression confirmed that age ≥75 years was associated with a significantly higher likelihood of NYHA III classification (OR = 3.754, 95% CI 1.543–9.134, *p* = 0.004) and more severe dyspnea (OR = 2.700, 95% CI 1.177–6.196, *p* = 0.019), whereas the association with reduced 6MWT did not reach statistical significance (OR = 2.346, 95% CI 0.939–5.862, *p* = 0.068).

A greater comorbidity burden was observed among patients with worse symptom severity and functional impairment. Each one-point increase in the Charlson Comorbidity Index was associated with approximately 55% higher odds of NYHA III classification (OR = 1.551, 95% CI 1.116–2.155, *p* = 0.009), while borderline associations were observed for reduced 6MWT (OR = 1.368, 95% CI 0.983–1.903, *p* = 0.063) and higher Borg scores (OR = 1.350, 95% CI 0.999–1.825, *p* = 0.051). Among comorbidities, atrial fibrillation consistently showed the strongest association with adverse outcomes. Patients with atrial fibrillation were more likely to belong to NYHA class III (OR = 3.800, 95% CI 1.554–9.293, *p* = 0.003), exhibit reduced exercise capacity (OR = 6.802, 95% CI 2.440–18.962, *p* < 0.001), and report severe dyspnea (OR = 2.750, 95% CI 1.224–6.177, *p* = 0.014). Similarly, pulmonary hypertension was associated with increased odds of NYHA III status (OR = 3.514, 95% CI 1.334–9.258, *p* = 0.011), reduced 6MWT performance (OR = 3.692, 95% CI 1.366–9.982, *p* = 0.010), and higher Borg scores (OR = 3.224, 95% CI 1.252–8.305, *p* = 0.015). Unexpectedly, arterial hypertension was associated with lower odds of NYHA class III (OR = 0.060, 95% CI 0.007–0.539, *p* = 0.012), reduced 6MWT performance (OR = 0.127, 95% CI 0.022–0.738, *p* = 0.022), and higher Borg score (OR = 0.093, 95% CI 0.010–0.828, *p* = 0.033) in the univariable analysis.

The presence of edema was strongly associated with worse clinical status. Peripheral edema was more prevalent among patients with NYHA III symptoms, reduced 6MWT performance, and higher Borg scores. Regression analysis showed that it increased the odds of NYHA III classification (OR = 4.725, 95% CI 1.034–21.601, *p* = 0.045) and severe dyspnea (OR = 7.412, 95% CI 1.634–33.613, *p* = 0.009). Increasing pitting edema severity demonstrated a dose-dependent relationship with NYHA class and exercise limitation. Compared with patients without pitting edema, grade 2 edema was associated with approximately tenfold higher odds of NYHA III status (OR = 10.238, 95% CI 2.182–48.017, *p* = 0.003) and twelvefold higher odds of reduced 6MWT performance (OR = 12.600, 95% CI 1.990–79.752, *p* = 0.007), while grade 3 edema showed even stronger associations (OR = 43.000 and OR = 75.001, respectively).

Body composition expressed as body fat percentage was significantly associated with NYHA III classification, reduced 6MWT performance, and severe dyspnea: obese BF% categories exhibited markedly increased odds of NYHA III status (OR = 16.762, 95% CI 2.038–137.830, *p* = 0.009), reduced exercise performance (OR = 13.391, 95% CI 1.621–110.596, *p* = 0.016), and severe dyspnea (OR = 7.037, 95% CI 1.781–27.807, *p* = 0.005). On the other hand, no significant association was observed between BMI or visceral fat obesity and any of the examined HFpEF functional status parameters.

Finally, inflammatory markers demonstrated the strongest and most consistent associations with disease severity. Elevated CRP (>5 mg/L) was associated with increased odds of NYHA III classification (OR = 5.038, 95% CI 1.853–13.699, *p* = 0.002), reduced 6MWT performance (OR = 6.811, 95% CI 2.152–21.553, *p* = 0.001), and severe dyspnea (OR = 9.833, 95% CI 3.657–24.434, *p* < 0.001). Elevated IL-6 (>7 pg/mL) exhibited the strongest associations, increasing the odds of NYHA III classification nearly 78-fold (OR = 77.625, 95% CI 16.285–370.019, *p* < 0.001) and reduced 6MWT performance more than 100-fold (OR = 112.000, 95% CI 14.039–893.506, *p* < 0.001), while also being associated with severe dyspnea (OR = 3.246, 95% CI 1.399–7.535, *p* = 0.006). Likewise, elevated NLR and PLR were consistently associated with all three adverse functional outcomes, supporting the role of systemic inflammation in HFpEF severity.

### 3.2. Distribution of IL6 Polymorphisms and Association with IL-6 Concentrations

Genetic analysis was successfully performed in a subset of 78 study patients. To evaluate potential selection bias, baseline demographic and clinical characteristics were compared between patients with and without available genotyping data. No statistically significant differences were observed between the two groups (Supplementary Table S2). Frequencies of minor alleles, genotypes, genotype groups (dominant and recessive genetic models), haplotypes, and diplotypes of *IL6* polymorphisms are presented in Supplementary Table S3. No significant deviation from Hardy–Weinberg equilibrium was observed for either polymorphism. The genotype distribution of rs1818879 (χ^2^ = 0.043, df = 1, *p* > 0.05) and rs1800795 (χ^2^ = 0.134, df = 1, *p* > 0.05) was consistent with expected frequencies under equilibrium conditions. Although rs1800795 and rs1818879 were in complete linkage disequilibrium (D′ = 1.0), the correlation between the variants was modest (r^2^ = 0.22), indicating limited predictability of one polymorphism from the other (Supplementary Figure S1).

IL-6 levels were not significantly associated with genotype distributions for either rs1800795 or rs1818879 when analyzed individually (Supplementary Table S4). However, in the recessive model of rs1800795, carriers of the C/C genotype exhibited significantly lower IL-6 levels compared to carriers of the G allele (OR = 0.192, 95% CI 0.040–0.924, *p* = 0.040). No statistically significant associations were detected in the dominant model or for the rs1818879 polymorphism within the limited genetic sample. Analysis of diplotypes did not reveal consistent patterns, although a borderline association was observed.

### 3.3. IL6 Polymorphism Association with Parameters of Functional Status

The distribution of *IL6* rs1800795 and rs1818879 genotypes, inheritance models, and diplotypes according to NYHA functional class, 6MWT performance, and Borg dyspnea score is presented in Table 2, Table 3 and Table 4. Initial analyses did not reveal significant differences in genotype frequencies across any of the functional status outcomes. This pattern was consistent after grouping genotypes according to dominant and recessive inheritance models, as well as in diplotype-based analyses. Thus, no statistically significant association between individual IL6 polymorphisms and functional status was detected within the limited sample size of the present study.

### 3.4. Multivariable Analysis of Functional Status Outcomes

Multivariable logistic regression analysis was performed to identify the best-fitting models for NYHA functional class, functional capacity assessed by the 6MWT, and Borg dyspnea score. To evaluate model stability, bootstrap validation was additionally performed (Table 5).

For both NYHA functional class and 6MWT performance, the best-fitting multivariable models identified NLR and the combined effect of elevated IL-6 levels and the dominant *IL6* rs1800795 genetic model as independent predictors of functional impairment. For NYHA functional class, the final model demonstrated good calibration (Hosmer–Lemeshow χ^2^(5) = 6.740, *p* = 0.241), explained 55.0% of outcome variability (Nagelkerke R^2^ = 0.550), and correctly classified 87.2% of patients. A similar pattern was observed for functional capacity assessed by the 6MWT. The final model was well calibrated (Hosmer–Lemeshow χ^2^(6) = 5.123, *p* = 0.528), explained 50.2% of outcome variability (Nagelkerke R^2^ = 0.502), and correctly classified 87.2% of cases. In both models, the unfavourable effect of elevated IL-6 was consistently more pronounced among carriers of the G/C+C/C genotype than among G/G homozygotes, increasing the odds of NYHA class III and reduced exercise capacity by approximately sixfold. NLR emerged as an independent predictor of functional impairment, being associated with an approximately 18-fold higher likelihood of NYHA class III and an 11-fold higher likelihood of reduced exercise capacity. For perceived dyspnea, the best-fitting model included age, pulmonary hypertension, and the combined effect of elevated IL-6 levels and the dominant *IL6* rs1800795 genetic model. The model demonstrated excellent calibration (Hosmer–Lemeshow χ^2^(7) = 2.298, *p* = 0.942), explained 25.8% of the variability in Borg scale categories (Nagelkerke R^2^ = 0.258), and correctly classified 71.8% of patients. Age over 75 years and pulmonary hypertension were associated with higher Borg scores, while carriers of the G/C+C/C genotype with elevated IL-6 levels exhibited greater susceptibility to severe dyspnea than G/G homozygotes.

The results of bootstrap resampling confirmed the robustness of the final multivariable models. The combined effect of elevated IL-6 levels and the dominant *IL6* rs1800795 genetic model, NLR, age, and pulmonary hypertension remained independently associated with NYHA functional class, 6MWT performance, and Borg dyspnea score after resampling in their respective models, indicating a stable effect across all assessed dimensions of functional status. Notably, CRP, which showed borderline significance in the original NYHA and 6MWT models (*p* = 0.080 and *p* = 0.093, respectively), achieved statistical significance following bootstrap validation (*p* = 0.030 and *p* = 0.040, respectively).

As a sensitivity analysis, the combined IL-6–rs1800795 predictor was additionally evaluated using IL-6 as a continuous variable instead of dichotomization. The results remained consistent with the primary analyses, with statistically significant associations observed for NYHA functional class (OR = 1.19, 95% CI 1.06–1.33, *p* = 0.003), reduced 6MWT performance (OR = 0.84, 95% CI 0.75–0.94, *p* = 0.003), and Borg dyspnea score (OR = 1.14, 95% CI 1.04–1.25, *p* = 0.004), supporting the robustness of the primary findings.

**Figure 1:**
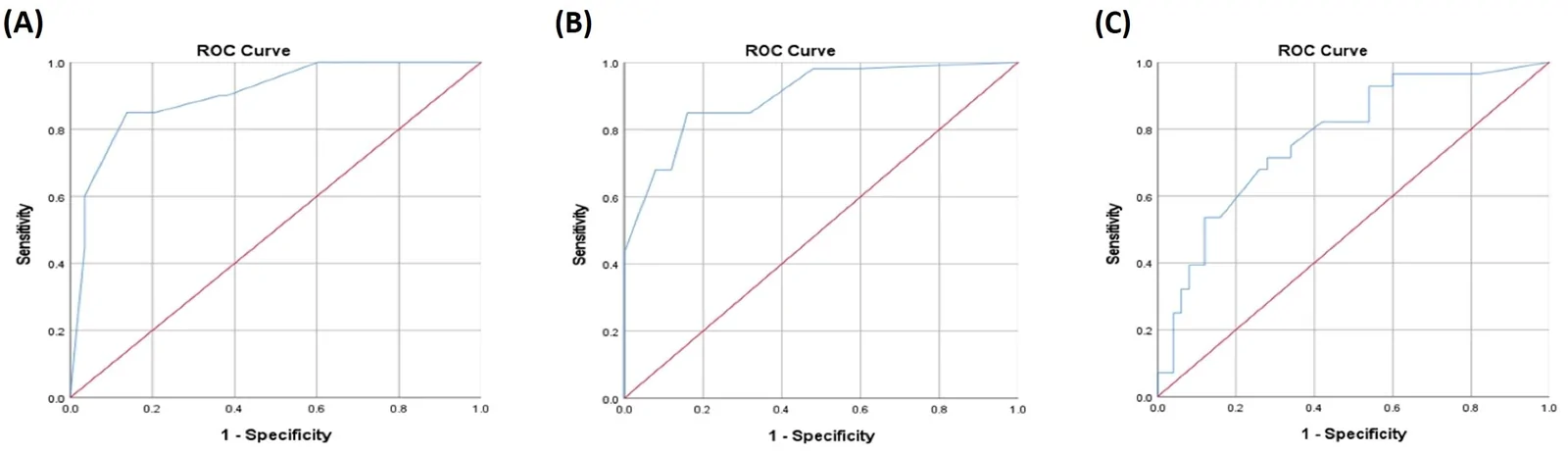
ROC curve for prediction of (**A**) NYHA class III, (**B**) reduced 6MWT performance, and (**C**) perceived exertional dyspnea as assessed by the Borg scale.

ROC curve analysis demonstrated robust predictive performance of the developed models across all functional status outcomes (Figure 1). The models demonstrated good-to-excellent discriminative performance across all functional status outcomes. The strongest discriminative ability was observed for NYHA class III (Figure 1A) (AUC = 0.902, 95%: CI 0.825–0.979, *p* < 0.001), closely followed by reduced functional capacity (Figure 1B) assessed by the 6MWT (AUC = 0.898, 95% CI: 0.829–0.968, *p* < 0.001). At the optimal probability thresholds, sensitivities and specificities exceeded 84% for both outcomes. Prediction of higher Borg scale categories (Figure 1C) was less accurate but remained statistically significant, with an AUC of 0.775 (95% CI: 0.667–0.882, *p* < 0.001), corresponding to a sensitivity of 71.4% and specificity of 72.0% at the optimal threshold.

## 4. Discussion

The present study investigated the relationship between parameters of systemic inflammation, *IL6* genetic variability, and functional status in patients with HFpEF. The principal finding was that inflammatory activation was consistently associated with symptom burden, reduced exercise capacity, and perceived exertional dyspnea. Most importantly, the combined effect of elevated IL-6 concentrations and the *IL6* rs1800795 genetic model emerged as an independent predictor of functional impairment across all examined domains, including NYHA class, predicted 6MWT performance, and Borg dyspnea score. Although IL6 polymorphisms were not independently associated with functional outcomes, their combined predictive contribution with elevated IL-6 was consistently associated with functional impairment, suggesting that the clinical consequences of inflammation in HFpEF may depend not only on the intensity of inflammatory activation, but also on the genetic context in which such activation occurs.

Inflammation is increasingly recognized as a central component of HFpEF pathophysiology. According to the comorbidity-driven paradigm proposed by Paulus and Tschöpe, aging and cardiometabolic disorders induce a chronic systemic inflammatory state that promotes coronary microvascular endothelial dysfunction, oxidative stress, reduced nitric oxide bioavailability, myocardial stiffening, interstitial fibrosis, and ultimately the development of HFpEF [26]. Subsequent studies and reviews have further expanded this concept, emphasizing that HFpEF is not merely a disorder of diastolic function, but a heterogeneous systemic syndrome in which inflammation, metabolic dysregulation, endothelial dysfunction, and peripheral abnormalities interact to determine symptom burden and functional limitation [11,27,28,29]. In line with this concept, our study demonstrated significant associations between inflammatory markers and all evaluated dimensions of functional impairment.

Insulin resistance represents another important mechanism linking metabolic dysfunction and chronic inflammation in HFpEF. Increasing evidence suggests that insulin resistance contributes to persistent low-grade inflammatory activation through enhanced production of pro-inflammatory cytokines, endothelial dysfunction, and oxidative stress, while inflammation may further aggravate metabolic abnormalities, creating a self-perpetuating cycle that promotes myocardial remodeling and functional impairment in HFpEF [11,27,28,29]. In this context, inflammatory indices may partially reflect the underlying metabolic-inflammatory disturbances characteristic of HFpEF. Although insulin resistance was not directly assessed in the present study, future investigations integrating metabolic and inflammatory markers may further improve phenotypic characterization and personalized risk stratification [2,27,28].

Among the investigated inflammatory markers, IL-6 occupies a particularly relevant position. IL-6 is a pleiotropic cytokine involved in immune activation, endothelial dysfunction, metabolic regulation, vascular inflammation, and myocardial remodeling [30,31]. In HFpEF, elevated IL-6 concentrations have been associated with greater symptom severity, poorer exercise capacity, worse patient-reported health status, and adverse clinical outcomes [32,33]. Recent data further support IL-6 as a potential upstream inflammatory mediator in HFpEF rather than merely a passive biomarker [32,33,34]. Our findings are consistent with this literature, as elevated IL-6 was strongly associated with adverse functional status. However, our results extend previous biomarker-focused studies by showing that the clinical impact of IL-6 may depend on the combined predictive contribution of inflammatory status and *IL6* genetic variability.

A particularly important finding of this study was the significant combined effect of circulating IL-6 levels and the rs1800795 polymorphism. While rs1800795 alone was not associated with NYHA class, 6MWT performance, or Borg dyspnea score, elevated IL-6 levels exerted different clinical effects depending on genetic background. This observation suggests that circulating IL-6 concentration alone may not fully reflect the biological consequences of IL-6 signaling. Instead, genetic variability may modify transcriptional regulation, downstream signaling efficiency, receptor responsiveness, tissue sensitivity, or compensatory inflammatory pathways, thereby influencing the clinical expression of inflammatory activation. The biological functionality of rs1800795 provides a plausible explanation for this observation. Namely, the rs1800795 polymorphism, also known as *IL6*-174G>C, is located within the promoter region of the *IL6* gene and has been extensively studied as a functional regulatory variant [35,36]. Experimental studies have demonstrated that this polymorphism can influence *IL6* promoter activity and cytokine production after inflammatory stimulation [35,36]. Several reports suggest that the C allele, particularly in the C/C genotype, may be associated with lower IL-6 transcription or lower circulating IL-6 concentrations, although the direction and magnitude of this effect vary across populations, experimental systems, and clinical conditions [12,35,36,37]. This variability is expected because promoter polymorphisms often exert context-dependent effects influenced by age, ethnicity, comorbidities, environmental exposures, metabolic state, and inflammatory stimuli [12,37]. In our cohort, the recessive model of rs1800795 was significantly associated with IL-6 concentrations: C/C homozygotes had lower odds of elevated IL-6 levels compared with carriers of the G allele. This finding is broadly consistent with studies reporting reduced IL-6 production among carriers of the C allele [35,36]. However, despite this association with lower IL-6 concentrations, rs1800795 showed no independent relationship with functional status. Instead, its clinical relevance became apparent through its combined effect with circulating IL-6. This apparent paradox is biologically meaningful. A genotype may influence baseline cytokine production, but the clinical consequences of cytokine elevation may depend on additional mechanisms, including receptor-mediated signaling, soluble receptor availability, trans-signaling activity, endothelial responsiveness, and target-tissue susceptibility [30,31,38]. Therefore, individuals with similar circulating IL-6 concentrations may experience different biological and clinical effects. This finding supports the interpretation that rs1800795 acts primarily as a modifier of inflammatory responsiveness rather than as an independent determinant of HFpEF severity. Under basal conditions, the polymorphism may have limited influence on clinical manifestations. However, once inflammatory activation occurs, genetic variability may modify how strongly elevated IL-6 translates into symptoms, exercise limitation, or dyspnea. Such a mechanism is consistent with the broader concept of gene–environment interactions, whereby genetic variants become clinically relevant only in the presence of specific biological or environmental triggers [39,40]. In the present study, systemic inflammation may represent precisely such a trigger.

The fact that the dominant model, rather than the recessive model, entered the final functional-status models deserves additional consideration. The recessive model was more informative for IL-6 concentrations, suggesting that C/C homozygosity was associated with a lower probability of elevated IL-6. In contrast, the dominant model captured the combined effect of IL-6 and rs1800795 in relation to NYHA class, 6MWT performance, and Borg score. This difference may indicate that the genetic architecture of IL-6 production is not identical to the genetic architecture of IL-6 clinical effects. In other words, one genetic model may better describe cytokine concentration, whereas another may better capture biological susceptibility to inflammation. Given the limited number of C/C homozygotes, the dominant model may also have provided greater statistical stability for evaluating the combined effect of elevated IL-6 levels and the rs1800795 genotype. Therefore, these findings should be interpreted cautiously and will require further confirmation.

In contrast to rs1800795, no statistically significant association of rs1818879 with IL-6 concentrations or functional outcomes was detected within the limited genetic sample. This may reflect differences in the biological relevance of the two polymorphisms. While rs1800795 directly affects the promoter region of *IL6*, rs1818879 is located in the 3′ region of the *IL6* locus, and its functionality is less clearly established. Available evidence suggests that rs1818879 may be involved in inflammatory regulation, or may act through linkage with other functional variants; however, direct data regarding its influence on circulating IL-6 are limited [41,42]. Therefore, the lack of association in our study is not unexpected. It may also reflect limited statistical power, particularly because genetic analyses were performed in a subset of the total study population.

Beyond IL-6-related genetic modulation, other inflammatory biomarkers emerged as important predictors of functional impairment. Elevated NLR was the strongest independent predictor in both the NYHA and 6MWT models, increasing the likelihood of advanced functional class and reduced exercise capacity. NLR is an integrative marker that reflects the balance between innate immune activation and adaptive immune suppression. Previous studies have shown that elevated NLR is associated with adverse outcomes in patients with heart failure, including mortality and hospitalization [43,44,45]. Its prognostic value has been demonstrated across heart failure phenotypes, including both reduced and preserved ejection fraction [43,44]. Because NLR is inexpensive, routinely available, and easy to calculate, it may represent a practical marker for identifying HFpEF patients with higher inflammatory burden and worse functional status. CRP also contributed to disease severity in our cohort. Although its effect was borderline in the original multivariable models, bootstrap validation confirmed its independent association with both NYHA class and 6MWT performance. CRP is a downstream marker of systemic inflammatory activity and has repeatedly been associated with HFpEF diagnosis, prognosis, and adverse outcomes [46,47,48]. Elevated CRP may reflect cumulative inflammatory burden arising from obesity, insulin resistance, endothelial activation, congestion, renal dysfunction, and comorbidity accumulation, all of which are common in HFpEF [27,28,46]. The emergence of statistical significance after bootstrap validation suggests that the contribution of CRP may be more stable than indicated by conventional regression alone.

Alongside inflammatory markers, comorbidities also showed relevant associations with functional impairment in HFpEF. In univariable analysis, atrial fibrillation was consistently associated with worse status across all three functional domains, including NYHA class III, reduced 6MWT performance, and higher Borg scores, while pulmonary hypertension also showed significant associations with all examined outcomes and remained an independent predictor of greater dyspnea burden in the final Borg model. These findings are clinically plausible, as atrial fibrillation may worsen functional capacity through loss of atrial contribution to ventricular filling, irregular ventricular response, and reduced cardiac reserve, whereas pulmonary hypertension contributes to exertional dyspnea through impaired pulmonary vascular recruitment and right ventricular–pulmonary arterial uncoupling [49,50].

A more unexpected finding was the inverse association between arterial hypertension and all three functional outcomes. This observation should not be interpreted as evidence of a protective effect of hypertension [51]. Given the observational design of the study, the very high prevalence of hypertension among patients with HFpEF, and the small number of normotensive individuals in our cohort, this association is likely explained by residual confounding, selection effects, or survivor bias rather than a true biological effect [52]. Therefore, this finding should be interpreted with considerable caution and requires confirmation in larger cohorts before any clinical conclusions can be drawn.

The strong predictive performance of the NYHA and 6MWT models further supports the clinical relevance of inflammatory activation. Both models achieved excellent discrimination, with AUC values approaching 0.90. Exercise intolerance is one of the defining manifestations of HFpEF and is increasingly understood as a consequence of both cardiac and peripheral abnormalities. While impaired ventricular relaxation, elevated filling pressures, and abnormal cardiac reserve contribute to reduced exercise capacity, skeletal muscle dysfunction, impaired mitochondrial energetics, endothelial dysfunction, pulmonary vascular abnormalities, and reduced peripheral oxygen extraction also play major roles [53,54,55,56]. Chronic inflammation may contribute to each of these mechanisms, providing a plausible biological explanation for the strong association between inflammatory markers and reduced 6MWT performance observed in our study. The 6MWT is widely used as a simple and clinically meaningful measure of functional capacity in heart failure [57]. Although cardiopulmonary exercise testing remains the gold standard for detailed assessment of exercise physiology, the 6MWT captures integrated functional performance and is particularly useful in older, comorbid HFpEF populations [22,57]. Our finding that inflammatory markers and the combined effect of elevated IL-6 levels and the rs1800795 genotype predicted reduced 6MWT performance supports the concept that inflammation contributes to real-world functional limitation, not only to biochemical or hemodynamic abnormalities.

The findings related to perceived exertional dyspnea provide additional insight into patient-reported symptom burden. In the final Borg model, age ≥75 years and pulmonary hypertension remained significant independent predictors alongside the combined effect of elevated IL-6 levels and the rs1800795 genotype. This finding is consistent with the known pathophysiology, as aging is associated with reduced cardiopulmonary reserve, skeletal muscle decline, impaired ventilatory efficiency, and increased comorbidity burden. Pulmonary hypertension, on the other hand, contributes directly to exertional breathlessness through increased pulmonary vascular resistance, impaired pulmonary vascular recruitment, right ventricular–pulmonary arterial uncoupling, and abnormal exercise hemodynamics [58,59]. Importantly, the persistence of the combined effect of elevated IL-6 levels and the rs1800795 genotype in this model suggests that inflammatory mechanisms may influence not only objective functional limitation but also patients’ subjective perception of exertional symptoms. The Borg model showed lower discriminative performance than the NYHA and 6MWT models, which is expected. Dyspnea perception is inherently subjective and is influenced by multiple factors, including age, pulmonary pressures, obesity, deconditioning, anxiety, anemia, respiratory comorbidities, congestion, and individual perception of breathlessness [58,59]. Therefore, Borg scale findings should be interpreted as complementary to NYHA and 6MWT results. Together, these three outcomes indicate that inflammation is relevant across physician-assessed, objectively measured, and patient-perceived domains of HFpEF severity.

A particularly noteworthy aspect of our findings is the consistency of the combined effect of elevated IL-6 levels and the rs1800795 genotype across all three functional-status domains. The same genetic-inflammatory signal remained significant in models assessing NYHA class, predicted 6MWT performance, and Borg dyspnea score. Moreover, bootstrap validation confirmed the stability of these associations. Bootstrap resampling is recommended for internal validation of predictive models because it provides a more realistic estimate of model stability and helps assess the extent to which findings may depend on sample-specific variation [60,61]. The persistence of the combined effect after resampling strengthens the credibility of this finding and reduces the likelihood that it represents a spurious statistical association.

From a clinical perspective, these findings support the concept of HFpEF as a heterogeneous inflammation-mediated syndrome. Traditional clinical variables alone often fail to fully explain the variability in symptoms and exercise limitation among HFpEF patients. Our results suggest that combining inflammatory biomarkers with genetic information may provide a more refined characterization of disease severity and help identify patients in whom inflammation plays a particularly important role. This approach is aligned with current efforts toward phenotype-specific and precision medicine strategies in HFpEF [62,63]. Although anti-inflammatory therapies in HFpEF remain investigational, improved identification of inflammation-driven phenotypes may facilitate better patient selection in future clinical trials.

Several limitations should be acknowledged. This was a single-center study with a moderate sample size, which may limit generalizability and reduce statistical power for detecting smaller effects. The reduced sample size for genetic analyses may have limited the power to detect independent associations between polymorphisms and clinical outcomes. Only selected *IL6* polymorphisms were analysed, and broader genetic profiling may provide further insight into HFpEF heterogeneity. In addition, circulating IL-6 was measured as a systemic biomarker and does not necessarily reflect local myocardial, vascular, skeletal muscle, or pulmonary inflammatory activity.

The modest overall sample size represents an important limitation, particularly because genetic analyses were available for only 78 participants. Some genotype–outcome subgroups contained very few patients, which reduced statistical power, increased the uncertainty of the effect estimates, and may have limited the ability to detect weaker associations. Therefore, the genetic findings, especially those derived from subgroup analyses and analyses of the combined effect of elevated IL-6 levels and the rs1800795 genotype, should be interpreted as exploratory and hypothesis-generating and require confirmation in larger, independent HFpEF cohorts.

Finally, longitudinal follow-up was not available, preventing assessment of whether the observed combined effect of elevated IL-6 levels and the rs1800795 genotype predicts future clinical deterioration or adverse outcomes.

## 5. Conclusions

Inflammatory activation in HFpEF is associated with both objective functional limitation and perceived symptom burden, while combined inflammatory and genetic information may provide additional predictive value. The observed combined effect of elevated IL-6 levels and the *IL6* rs1800795 polymorphism supports the concept of HFpEF as a heterogeneous inflammation-mediated syndrome and suggests that integrating inflammatory and genetic profiling may improve future personalized risk stratification approaches.

## Figures and Tables

**Table 1:** Demographic, clinical, anthropometric, and laboratory characteristics of HFpEF patients stratified by NYHA class, predicted 6MWT distance, and Borg score.

Variable, n (%)	NYHA II n = 81	NYHA III n = 29	*p*	Preserved 6MWT n = 85	Reduced 6MWT n = 25	*p*	Borg 0–4 n = 72	Borg 5–10 n = 38	*p*
**DEMOGRAPHICS**									
Female sex	42 (51.9)	16 (55.2)	0.759	48 (56.5)	10 (40.0)	0.147	36 (50.0)	22 (57.9)	0.430
Age ≥75 years	18 (22.2)	13 (44.8)	**0.003**	24 (28.2)	12 (48.0)	0.064	18 (25.0)	18 (47.4)	**0.017**
**COMORBIDITIES**									
CCI, median (IQR)	3 (3–4)	4 (3–6)	**0.006**	4 (3–4)	4 (3–6)	0.053	3 (3–4)	4 (3–5)	**0.037**
Arterial hypertension	80 (98.8)	24 (82.8)	**0.005**	83 (97.6)	21 (84.0)	**0.008**	71 (98.6)	33 (86.8)	**0.018**
Hyperlipidemia	61 (75.3)	19 (65.5)	0.310	66 (77.6)	14 (56.0)	0.033	56 (77.8)	24 (63.2)	0.102
Diabetes mellitus	37 (45.7)	13 (44.8)	0.937	40 (47.1)	10 (40.0)	0.533	32 (44.4)	18 (47.4)	0.770
Atrial fibrillation	27 (33.3)	19 (65.5)	**0.003**	27 (31.8)	19 (76.0)	**<0.001**	24 (33.3)	22 (57.9)	0.013
Anemia	18 (22.2)	11 (37.9)	0.099	19 (22.4)	10 (40.0)	0.078	17 (23.6)	12 (31.6)	0.367
Chronic kidney disease	16 (19.8)	11 (37.9)	0.077	18 (21.2)	9 (36.0)	0.130	15 (20.8)	12 (31.6)	0.213
COPD	4 (4.9)	5 (17.2)	0.052	4 (4.7)	5 (20.0)	**0.027**	3 (4.2)	6 (15.8)	**0.034**
Pulmonary hypertension	12 (14.8)	11 (37.9)	**0.015**	13 (15.3)	10 (40.0)	**0.008**	10 (13.9)	13 (34.2)	**0.013**
Hypothyroidism	10 (12.3)	7 (24.1)	0.132	12 (14.1)	5 (20.0)	0.474	9 (12.5)	8 (21.1)	0.238
**PRESENCE OF EDEMA**									
Peripheral edema	60 (74.1)	27 (93.1)	**0.031**	63 (74.1)	24 (96.0)	**0.018**	51 (70.8)	36 (94.7)	**0.003**
Pitting edema grade	0:21 (25.9)	0:2 (6.9)	**<0.001**	0:22 (25.9)	0:1 (4.0)	**<0.001**	0:21 (29.2)	0:2 (5.3)	**<0.001**
	1:50 (61.7)	1:13 (44.8)		1:51 (60.0)	1:12 (48.0)		1:42 (58.3)	1:21 (55.3)	
	2:10 (12.3)	2:12 (41.4)		2:12 (14.1)	2:10 (40.0)		2:9 (12.5)	2:13 (34.2)	
	3:0 (0.0)	3:2 (6.9)		3:0 (0.0)	3:2 (8.0)		3:0 (0.0)	3:2 (5.3)	
**BODY COMPOSITION**									
BMI category	H:14 (17.3);	H:8 (27.6);	0.370	H:14 (16.5);	H:8 (32.0);	0.186	H:13 (18.1);	H:9 (23.7);	0.711
	OW:22 (27.2);	OW:5 (17.2);		OW:23 (27.1);	OW:4 (16.0);		OW:19 (26.4);	OW:8 (21.1);	
	O:45 (55.6)	O:16 (55.2)		O:48 (56.5)	O:13 (52.0)		O:40 (55.6)	O:21 (55.3)	
BF% category	H:22 (27.2);	H:1 (3.4);	**0.003**	H:22 (25.9);	H:1 (4.0);	**0.009**	H:20 (27.8);	H:3 (7.9);	**0.009**
	OW:38 (46.9);	OW:12 (41.4);		OW:40 (47.1);	OW:10 (40.0);		OW:34 (47.2);	OW:16 (42.1);	
	O:21 (25.9)	O:16 (55.2)		O:23 (27.1)	O:14 (56.0)		O:18 (25.0)	O:19 (50.0)	
VF category	H:8 (9.9);	H:1 (3.4);	**<0.001**	H:8 (9.4);	H:1 (4.0);	**<0.001**	H:7 (9.7);	H:2 (5.3);	**0.040**
	OW:52 (64.2);	OW:6 (20.7);		OW:53 (62.4);	OW:5 (20.0);		OW:43 (59.7);	OW:15 (39.5);	
	O:21 (25.9)	O:22 (75.9)		O:24 (28.2)	O:19 (76.0)		O:22 (30.6)	O:21 (55.3)	
WC category	L:10 (12.3);	L:5 (17.2);	0.535	L:9 (10.6);	L:6 (24.0);	0.102	L:8 (11.1);	L:7 (18.4);	0.288
	H:71 (87.7)	H:24 (82.8)		H:76 (89.4)	H:19 (76.0)		H:64 (88.9)	H:31 (81.6)	
**SELECTED INFLAMMATORY MARKERS**									
CRP > 5 vs. ≤5 mg/L	35 (43.2)	23 (79.3)	**<0.001**	37 (43.5)	21 (84.0)	**<0.001**	31 (43.1)	27 (71.1)	**0.005**
IL-6 > 7 vs. ≤7 pg/mL	12 (14.8)	27 (93.1)	**<0.001**	15 (17.6)	24 (96.0)	**<0.001**	13 (18.1)	26 (68.4)	**<0.001**
NLR ≥ 3 vs. <3	27 (33.3)	23 (79.3)	**<0.001**	29 (34.1)	21 (84.0)	**<0.001**	25 (34.7)	25 (65.8)	**0.002**
PLR ≥ 150 vs. <150	23 (28.4)	18 (62.1)	**<0.001**	26 (30.6)	15 (60.0)	**0.008**	21 (29.2)	20 (52.6)	**0.016**

Note: Values in bold indicate statistically significant results; Abbreviations: CCI—Charlson Comorbidity Index; COPD—chronic obstructive pulmonary disease; BMI—body mass index; BF—body fat; VF—visceral fat; WC—waist circumference; H—healthy; OW—overweight; O—obese; L—low; CRP—C-reactive protein; IL-6—interleukin-6; NLR—neutrophil-to-lymphocyte ratio; PLR—platelet-to-lymphocyte ratio.

**Table 2:** Distribution of *IL6* gene polymorphisms according to NYHA functional class.

Variable	NYHA II	NYHA III	χ ^2^ *p*	Crude OR (95% CI)
	n = 58	n = 20		
	n (%)			
rs1800795				
Genotype				
G/G	19 (32.8)	8 (40.0)	3.062 0.216	Ref.
G/C	26 (44.8)	11 (55.0)		1.005 [0.339; 2.977]
C/C	13 (22.4)	1 (5.0)		0.183 [0.020; 1.641]
Recessive model				
G/G+G/C	44 (75.9)	19 (95.0)	3.507 0.098	Ref.
C/C	14 (24.1)	1 (5.0)		0.165 [0.020; 1.349]
Dominant model				
G/G	19 (32.8)	8 (40.0)	0.345 0.557	Ref.
G/C+C/C	39 (67.2)	12 (60.0)		1.368 [0.479; 3.908]
rs1818879				
Genotype				
G/G	34 (58.6)	11 (55.0)	1.856 0.395	Ref. †
G/A	20 (34.5)	9 (45.0)		1.391 [0.492; 3.934]
A/A	4 (6.9)	0 (0.0)		0.333 [0.016; 6.675]
Recessive model				
G/G+G/A	93.1 (54)	100.0 (20)	1.454 0.567	Ref. †
A/A	6.9 (4)	0.0 (0)		0.060 [0.001; 3.411]
Dominant model				
G/G	58.6 (34)	55.0 (11)	0.080 0.777	Ref.
G/A+A/A	41.4 (24)	45.0 (9)		1.159 [0.416; 3.228]
Diplotype rs1818879–rs1800795				
A-G/A-G	4 (6.9)	0 (0.0)	6.120 0.295	Ref. †
A-G/G-C	10 (17.2)	5 (25.0)		0.473 [0.008; 27.938]
A-G/G-G	10 (17.2)	4 (20.0)		1.631 [0.029; 89.282]
G-C/G-C	13 (22.4)	1 (5.0)		1.526 [0.027; 83.700]
G-C/G-G	16 (27.6)	6 (30.0)		1.526 [0.027; 83.700]
G-G/G-G	5 (8.6)	4 (20.0)		2.368 [0.043; 128.359]

“†”—Firth logistic regression was used due to zero-cell frequencies. Abbreviations: 95% CI, 95% confidence interval for the estimated OR; OR, odds ratio; *p*, probability; χ^2^, Chi-square statistics for the degree of freedom of 1 (df = 1).

**Table 3:** Distribution of *IL6* polymorphisms according to 6MWT performance.

Variable	Predicted 6MWT Distance		χ ^2^ *p*	Crude OR (95% CI)
	Preserved	Reduced		
	n = 61	n = 17		
	n (%)			
rs1800795				
Genotype				
G/G	21 (34.4)	6 (35.3)	2.360 0.307	Ref.
G/C	27 (44.3)	10 (58.8)		0.269 [0.029; 2.497]
C/C	13 (21.3)	1 (5.9)		0.208 [0.024; 1.800]
Recessive model				
G/G+G/C	47 (77.0)	16 (94.1)	2.494 0.114	Ref.
C/C	14 (23.0)	1 (5.9)		0.210 [0.026; 1.725]
Dominant model				
G/G	21 (34.4)	6 (35.3)	0.004 0.947	Ref.
G/C+C/C	40 (65.6)	11 (64.7)		0.963 [0.312; 2.968]
rs1818879				
Genotype				
G/G	33 (54.1)	12 (70.6)	2.094 0.351	Ref. †
G/A	24 (39.3)	5 (29.4)		0.601 [0.194; 1.862]
A/A	4 (6.6)	0 (0.0)		0.297 [0.014; 5.939]
Recessive model				
G/G+G/A	56 (91.8)	17 (100.0)	1.489 0.222	Ref. †
A/A	5 (8.2)	0 (0.0)		0.293 [0.0154; 5.575]
Dominant model				
G/G	33 (54.1)	12 (70.6)	1.481 0.224	Ref.
G/A+A/A	28 (45.9)	5 (29.4)		0.491 [0.154; 1.564]
Diplotype rs1818879–rs1800795				
A-G/A-G	4 (6.6)	0 (0.0)	7.375 0.194	Ref. †
A-G/G-C	12 (19.7)	3 (17.6)		2.519 [0.107; 58.983]
A-G/G-G	12 (19.7)	2 (11.8)		1.799 [0.071; 45.135]
G-C/G-C	13 (21.3)	1 (5.9)		0.999 [0.034; 29.187]
G-C/G-G	15 (24.6)	7 (41.2)		4.354 [0.206; 91.864]
G-G/G-G	5 (8.2)	4 (23.5)		7.363 [0.3071; 76.411]

“†”—Firth logistic regression was used due to zero-cell frequencies. Abbreviations: 95% CI, 95% confidence interval for the estimated OR; OR, odds ratio; *p*, probability; χ^2^, Chi-square statistics for the degree of freedom of 1 (df = 1).

**Table 4:** Distribution of *IL6* polymorphisms according to Borg scale.

Variable	Borg Scale Categories		χ ^2^ *p*	Crude OR (95% CI)
	0–4	5–10		
	n = 50	n = 28		
	n (%)			
rs1800795				
Genotype				
G/G	18 (36.0)	9 (32.1)	0.75 0.687	Ref.
G/C	22 (44.0)	15 (53.6)		1.250 [0.306; 5.114]
C/C	10 (20.0)	4 (14.3)		1.705 [0.450; 6.460]
Recessive model				
G/G+G/C	39 (78.0)	24 (85.7)	0.688 0.407	Ref.
C/C	11 (22.0)	4 (14.3)		1.692 [0.484; 5.920]
Dominant model				
G/G	18 (36.0)	9 (32.1)	0.118 0.731	Ref.
G/C+C/C	32 (64.0)	19 (67.9)		0.842 [0.316; 2.246]
rs1818879				
Genotype				
G/G	28 (56.0)	17 (60.7)	0.301 0.86	Ref.
G/A	19 (38.0)	10 (35.7)		1.821 [0.175; 18.947]
A/A	3 (6.0)	1 (3.6)		1.579 [0.145; 17.218]
Recessive model				
G/G+G/A	46 (92.0)	27 (96.4)	0.587 0.444	Ref.
A/A	4 (8.0)	1 (3.6)		2.348 [0.249; 22.103]
Dominant model				
G/G	28 (56.0)	17 (60.7)	0.163 0.686	Ref.
G/A+A/A	22 (44.0)	11 (39.3)		1.214 [0.473; 3.114]
Diplotype rs1818879–rs1800795				
A-G/A-G	3 (6.0)	1 (3.6)	4.076 0.539	Ref.
A-G/G-C	8 (16.0)	7 (25.0)		0.267 [0.019; 3.653]
A-G/G-G	11 (22.0)	2 (10.7)		0.700 [0.133; 3.684]
G-C/G-C	10 (20.0)	4 (14.3)		0.218 [0.035; 1.364]
G-C/G-G	14 (28.0)	8 (28.6)		0.320 [0.055; 1.847]
G-G/G-G	4 (8.0)	5 (17.9)		0.457 [0.095; 2.210]

Abbreviations: 95% CI, 95% confidence interval for the estimated OR; OR, odds ratio; *p*, probability; χ^2^, Chi-square statistics for the degree of freedom of 1 (df = 1).

**Table 5:** Independent predictors of NYHA class, 6MWT performance, and Borg dyspnea score identified by multivariable logistic regression with bootstrap validation.

						Bootstrapping Analysis	
Predictors	B	S.E.	Wald	*p*	OR [95% CI]	*p*	95% CI
**NYHA class**							
CRP ^a^	1.327	0.757	3.072	0.080	3.769 [0.855; 16.616]	**0.030**	0.068; 3.590
NLR ^a^	2.870	0.863	11.067	**0.001**	17.635 [3.251; 95.651]	**0.002**	1.410; 22.224
Combined elevated IL-6 and dominant rs1800795 genotype ^b^	1.804	0.731	6.094	**0.014**	6.076 [1.450; 25.452]	**0.006**	0.308; 4.047
Constant	−4.335	1.006	18.584	<0.0001	0.013		
**Predicted 6MWT distance**							
CRP ^a^	1.345	0.801	2.818	0.093	3.839 [0.798; 18.467]	**0.040**	−0.243; 19.933
NLR ^a^	2.406	0.863	7.778	**0.005**	11.085 [2.044; 60.112]	**0.004**	0.968; 21.284
Combined elevated IL-6 and dominant rs1800795 genotype ^b^	1.879	0.718	6.859	**0.009**	6.548 [1.604; 26.725]	**0.008**	0.487; 4.037
Constant	−4.384	1.033	18.013	<0.0001	0.012		
**Borg scale**							
Age ^a^	1.245	0.571	4.747	**0.029**	3.472 [1.133; 10.641]	**0.014**	0.157; 2.609
Pulmonary hypertension ^a^	1.638	0.625	6.874	**0.009**	5.145 [1.512; 17.504]	**0.009**	0.379; 3.306
Combined elevated IL-6 and dominant rs1800795 genotype ^b^	0.152	0.07	4.749	**0.029**	1.165 [1.015; 1.336]	**0.041**	0.012; 0.342
Constant	−2.030	0.506	16.095	<0.0001	0.0131		

Note: Values in bold indicate statistically significant results. Abbreviations: 95% CI, 95% confidence interval for the estimated OR; B, the regression coefficient; OR, odds ratio; *p*, probability; S.E., standard error; Wald χ^2^, Wald test statistics for the degree of freedom of 1 (df = 1). ^a^ Reference category defined as: CRP ≤ 5 mg/L, IL-6 ≤ 7 pg/mL, NLR < 3, age <75 years, and no pulmonary hypertension; ^b^ The combined IL-6–rs1800795 variable was coded as 1 for the joint presence of IL-6 > 7 pg/mL and the G/C+C/C genotype group and as 0 for all other combinations.

## Data Availability

The data presented in this study are available on request from the corresponding author.

## Supplementary Materials

The following supporting information can be downloaded at: https://www.mdpi.com/article/10.3390/biomedicines14081783/s1, Table S1. Univariable logistic regression analysis of factors associated with NYHA class III, reduced 6MWT distance, and higher Borg score in HFpEF patients. Table S2. Baseline characteristics of patients with and without available IL6 genotyping data. Table S3. Allelic, genotypic, haplotypic, and diplotypic frequencies of *IL6* polymorphisms rs1800795 and rs1818879 and their genetic inheritance models in the study population. Figure S1. Linkage disequilibrium across analyzed *IL6* SNPs visualized by the Haploview software and displayed as pairwise (A) D’, with empty cell corresponding to D’ = 100% or (B) r2. Table S4. IL-6 levels according to *IL6* gene polymorphisms.

## Abbreviations

The following abbreviations are used in this manuscript:

6MWT	Six-minute walk test
AUC	Area under the curve
BF%	Body fat percentage
BMI	Body Mass Index
CCI	Charlson Comorbidity Index
CRP	C-reactive protein
HF	Heart failure
HfpEF	Heart failure with preserved ejection fraction
IL-6	Interleukin-6
IQR	Interquartile Range
NLR	Neutrophil-to-lymphocyte ratio
NYHA	New York Heart Association
PLR	Platelet-to-lymphocyte ratio
ROC	Receiver operating characteristic
VF	Visceral fat
WC	Waist circumference
