# Supplementary material for: Inflammatory and Genetic Determinants of Functional Status in HFpEF: The Role of Circulating IL-6 and IL6 Polymorphisms
Source: Biomedicines. 2026 Aug 7;14(8):1783. doi: 10.3390/biomedicines14081783 (PMC13509477; doi:10.3390/biomedicines14081783)
Supplement: Supplementary file 1 [file biomedicines-14-01783-s001.zip › biomedicines-4448857-supplementary.pdf]

Supplementary Materials

Supplementary Table S1. Univariable logistic regression analysis of factors associated with NYHA class III, reduced 6MWT distance, and higher Borg score in HFpEF patients

Variable, n (%)	NYHA III OR [95% CI]	*p*	Reduced 6MWT OR [95% CI]	*p*	Borg 5–10 OR [95% CI]	*p*
**DEMOGRAPHICS**						
Female sex	1.143 [0.488; 2.679]	*0.759*	0.514 [0.207; 1.274]	*0.151*	1.375 [0.622; 3.037]	*0.431*
Age ≥75 years	3.754 [1.543; 9.134]	***0.004***	2.346 [0.939; 5.862]	*0.068*	2.700 [1.177; 6.196]	***0.019***
**COMORBIDITIES**						
CCI	1.551 [1.116; 2.155]	***0.009***	1.368 [0.983; 1.903]	*0.063*	1.350 [0.999; 1.825]	*0.051*
Arterial hypertension	0.060 [0.007; 0.539]	***0.012***	0.127 [0.022; 0.738]	***0.022***	0.093 [0.010; 0.828]	***0.033***
Hyperlipidemia	0.623 [0.249; 1.559]	*0.312*	0.366 [0.143; 0.938]	***0.036***	0.490 [0.207; 1.160]	*0.105*
Diabetes mellitus	0.966 [0.412; 2.266]	*0.937*	0.750 [0.303; 1.857]	*0.534*	1.125 [0.511; 2.475]	*0.770*
Atrial fibrillation	3.800 [1.554; 9.293]	***0.003***	6.802 [2.440; 18.962]	***<0.001***	2.750 [1.224; 6.177]	***0.014***
Anemia	2.139 [0.857; 5.340]	*0.103*	0.432 [0.167; 1.115]	*0.083*	0.670 [0.279; 1.605]	*0.369*
Chronic kidney disease	2.483 [0.981; 6.282]	*0.055*	2.094 [0.795; 5.514]	*0.135*	1.754 [0.721; 4.269]	*0.216*
COPD	4.010 [0.997; 16.137]	*0.051*	5.062 [1.245; 20.589]	***0.023***	4.312 [1.014; 18.346]	***0.048***
Pulmonary hypertension	3.514 [1.334; 9.258]	***0.011***	3.692 [1.366; 9.982]	***0.010***	3.224 [1.252; 8.305]	***0.015***
Hypothyroidism	2.259 [0.769; 6.638]	*0.138*	1.521 [0.479; 4.825]	*0.477*	1.867 [0.655; 5.318]	*0.243*
**PRESENCE OF EDEMA**						
Peripheral edema	4.725 [1.034; 21.601]	***0.045***	0.119 [0.015; 0.935]	***0.043***	7.412 [1.634; 33.613]	***0.009***
Pitting edema grade 1 vs 0†	2.299 [0.544; 9.711]	*0.257*	3.640 [0.623; 21.258]	*0.151*	2.702 [0.52; 140.110]	*0.622*
Pitting edema grade 2 vs 0†	10.238 [2.182; 48.017]	***0.003***	12.600 [1.990; 79.752]	***0.007***	0.957 [0.018; 50.342]	*0.983*
Pitting edema grade 3 vs 0†	43.000 [1.575; 1173.768]	***0.026***	75.001 [2.364; 2379.248]	***0.014***	0.106 [0.001; 6.621]	*0.288*
**BODY COMPOSITION**						
BMI: Overweight vs Healthy	0.398 [0.108; 1.464]	*0.165*	0.304 [0.077; 1.200]	*0.089*	0.608 [0.186; 1.990]	*0.411*
BMI: Obese vs Healthy	0.622 [0.220; 1.759]	*0.371*	0.474 [0.164; 1.372]	*0.169*	0.785 [0.279; 2.063]	*0.588*
BF%: Overweight vs Healthy	6.947 [0.845; 57.103]	*0.071*	5.500 [0.0660; 45.841]	*0.115*	3.317 [0.812; 12.116]	*0.097*
BF%: Obese vs Healthy	16.762 [2.038; 137.830]	***0.009***	13.391 [1.621; 110.596]	***0.016***	7.037 [1.781; 27.807]	***0.005***

VF: Overweight vs Healthy	0.923 [0.098; 8.706]	*0.944*	0.755 [0.078; 7.321]	*0.808*	1.221 [0.228;3.536]	*0.816*
VF: Obese vs Healthy	8.381 [0.964; 72.901]	*0.054*	6.333 [0.727; 55.150]	*0.095*	3.341 [0.622; 17.951]	*0.160*
WC: High vs Low	0.676 [0.210; 2.176]	*0.512*	0.375 [0.119; 1.183]	*0.094*	0.554 [0.184; 1.665]	*0.293*
**SELECTED INFLAMMATORY MARKERS**						
CRP >5 vs ≤ 5 mg/L	5.038 [1.853; 13.699]	***0.002***	6.811 [2.152; 21.553]	***0.001***	9.833 [3.657; 24.434]	***<0.001***
IL-6 >7 vs ≤ 7 pg/mL	77.625 [16.285; 370.019]	***<0.001***	112.000 [14.039; 893.506]	***<0.001***	3.246 [1.399; 7.535]	***0.006***
NLR ≥3 vs <3	7.667 [2.792; 21.065]	***<0.001***	10.138 [3.180; 32.322]	***<0.001***	3.615 [1.581; 8.269]	***0.002***
PLR ≥150 vs <150	4.126 [1.691; 10.070]	***0.002***	3.404 [1.352; 8.572]	***0.009***	2.968; [1.195; 6.094]	***0.017***

"†" - Firth logistic regression was used due to zero-cell frequencies; Note: Values in bold indicate statistically significant results; Abbreviations: CCI – Charlson Comorbidity Index; COPD – chronic obstructive pulmonary disease; BMI – body mass index; BF – body fat; VF – visceral fat; WC – waist circumference; CRP – C-reactive protein; IL-6 – interleukin-6; NLR – neutrophil-to-lymphocyte ratio; PLR – platelet-to-lymphocyte ratio; 95% CI - 95% confidence interval for the estimated OR; OR - odds ratio; p - probability.

Supplementary Table S2. Baseline characteristics of patients with and without available *IL6* genotyping data

**Variable**	**Genotyped (n=78)**	**Not genotyped (n=32)**	***p***
	**n (%) or median (IQR)**		
Age ≥75 years	25 (32.1)	11 (30.6)	0.814
Female sex	41 (52.6)	17 (53.1)	0.957
CCI	4.0 (3.0-5.0)	4.0 (3.0-5.0)	0.157
Arterial hypertension	74 (94.9)	30 (93.8)	0.814
Atrial fibrillation	33 (42.3)	13 (40.6)	0.871
COPD	5 (6.4)	4 (12.5)	0.290
Pulmonary hypertension	19 (24.4)	4 (12.5)	0.165
CRP >5 mg/L	41 (70.7)	17 (53.1)	0.957
IL-6 >7 pg/mL	27 (34.6)	12 (37.5)	0.774
NLR ≥3	35 (44.9)	15 (46.9)	0.848
PLR ≥150	27 (34.6)	14 (43.8)	0.368

p - probability

Supplementary Table S3. Allelic, genotypic, haplotypic, and diplotypic frequencies of *IL6* polymorphisms rs1800795 and rs1818879 and their genetic inheritance models in the study population.

**rs1800795**		**n/N (%)**	**95% CI**
Allele			
	G	91/156 (58.3%)	50.487–65.779
	C	65/156 (41.7%)	34.221–49.513
Genotype			
	G/G	27/78 (34.6%)	25.005–45.670
	G/C	37/78 (47.6%)	36.737–58.375
	C/C	14/78 (17.9%)	11.004–27.903
Recessive model			
	G/G+G/C	64/78 (82.1%)	70.665–87.985
	C/C	14/78 (17.9%)	12.015–29.335
Dominant model			
	G/G	27/78 (34.6%)	25.005–45.670
	G/C+C/C	51/78 (65.4%)	54.330–74.995
**rs1818879**			
Allele			
	G	119/156 (76.3%)	69.026–82.275
	A	37/156 (23.7%)	17.725–30.974
Genotype			
	G/G	45/78 (57.7%)	46.621–68.041
	G/A	29/78 (37.2%)	27.293–48.269
	A/A	4/78 (5.1%)	2.012–12.457
Recessive model			
	G/G+G/A	74/78 (94.9%)	85.856–97.231
	A/A	4/78 (5.1%)	2.769–14.144
Dominant model			
	G/G	45/78 (57.7%)	46.621–68.041
	G/A+A/A	33/78 (42.3%)	31.959–53.379
**rs1818879 - rs1800795**			
Haplotype			
	A-G	37/156 (23.7%)	17.725–30.974
	G-C	65/156 (41.7%)	34.221–49.513
	G-G	54/156 (34.6%)	27.601–42.370
Diplotype			
	A-G/A-G	4/78 (5.1%)	2.012–12.457
	A-G/G-C	15/78 (19.2%)	12.015–29.335
	A-G/G-G	14/78 (17.9%)	11.004–27.903
	G-C/G-C	14/78 (17.9%)	11.004–27.903
	G-C/G-G	22/78 (28.2%)	19.425–39.031
	G-G/G-G	9/78 (11.5%)	6.190–20.497

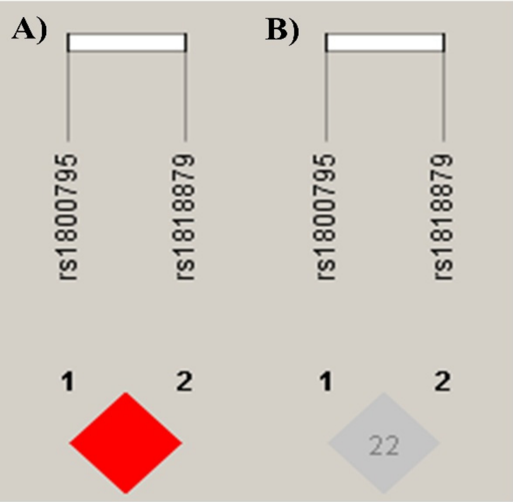

Supplementary Figure S1. Linkage disequilibrium across analyzed *IL6* SNPs visualized by the Haploview software and displayed as pairwise A) D′, with empty cell corresponding to D′=100% or B) r2 .

Supplementary Table S4. IL-6 levels according to *IL6* gene polymorphisms

		**IL-6**				
		**≤7 pg/mL n (%)**	**>7 pg/mL n (%)**	***P****	**OR [95%CI]**	***p***
**rs1800795**						
**Genotype**						
	G/G	19 (70.4)	8 (29.6)		*Ref.*	1
	G/C	20 (54.1)	17 (45.9)	*0.084*	2.111 [0.756; 5.899]	*0.154*
	C/C	12 (85.7)	2 (14.3)		0.333 [0.061; 1.820]	*0.205*
**Recessive model**						
	G/G+G/C	38 (59.4)	26 (40.6)	*0.072*	*Ref.*	1
	C/C	12 (85.7)	2 (14.3)		0.192 [0.040; 0.924]	***0.040***
**Dominant model**						
	G/G	19 (70.4)	8 (29.6)	*0.619*	*Ref.*	1
	G/C+C/C	32 (62.7)	19 (37.3)		1.400 [0.528; 3.713]	*0.499*
**rs1818879**						
**Genotype**						
	G/G	26 (57.8)	19 (42.2)		*Ref.*†	1
	G/A	21 (72.4)	8 (27.6)	*0.142*	0.537 [0.200; 1.440]	*0.217*
	A/A	4 (100.0)	0 (0.0)		0.150 [0.007; 2.971]	*0.214*
**Recessive model**						
	G/G+G/A	47 (63.5)	27 (36.5)	*0.292*	*Ref.*†	
	A/A	4 (100.0)	0 (0.0)		0.153 [0.008; 2.888]	*0.211*
**Dominant model**						
	G/G	26 (57.8)	19 (42.2)	*0.099*		
	G/A+A/A	25 (75.8)	8 (24.2)		0.543 [0.211; 1.401]	*0.207*
**Diplotype**						
	A-G/A-G	4 (100.0)	0 (0.0)	***0.045***	*Ref.*†	1

A-G/G-C	10 (66.7)	5 (33.3)	4.714 [0.212; 104.492]	*0.327*
A-G/G-G	11 (78.6)	3 (21.4)	2.739 [0.116; 64.384]	*0.532*
G-C/G-C	12 (85.7)	2 (14.3)	1.799 [0.071; 45.134]	*0.721*
G-C/G-G	10 (45.5)	12 (54.5)	10.714 [0.515; 222.811]	*0.126*
G-G/G-G	4 (44.4)	5 (55.6)	10.999 [0.459; 263.526]	*0.140*

"†" - Firth logistic regression was used due to zero-cell frequencies; * Comparisons among groups were performed using Chi-square test. Abbreviations: 95% CI - 95% confidence interval for the estimated OR; OR - odds ratio; p - probability.
